# Unlocking the potential of GLP-1 receptor agonists in ocular therapeutics: from molecular pathways to clinical impact

**DOI:** 10.3389/fphar.2025.1618079

**Published:** 2025-07-24

**Authors:** Zhixuan Xie, Zuyi Yang, Dianzhe Tian, Youxin Chen

**Affiliations:** ^1^ Department of Ophthalmology, Peking Union Medical College Hospital, Chinese Academy of Medical Sciences, Beijing, China; ^2^ Key Lab of Ocular Fundus Diseases, Chinese Academy of Medical Sciences, Beijing, China; ^3^ Eight-Year Medical Doctor Program, Chinese Academy of Medical Sciences and Peking Union Medical College, Beijing, China

**Keywords:** glucagon-like peptide-1 receptor agonists, ocular diseases, glaucoma, diabetic retinopathy, mechanism

## Abstract

Glucagon-like peptide-1 receptor agonists (GLP-1RAs), initially developed for diabetes and obesity, exhibit pleiotropic effects—including anti-inflammatory, antioxidant, and neuroprotective properties—that position them as transformative candidates for ocular therapeutics. Ocular diseases such as glaucoma and diabetic retinopathy (DR) are driven by multifactorial pathogenesis. Current therapies often target specific pathways or symptoms, and the potential effect of GLP-1RAs on the eye is still debated. The latest research and trials on the effect of GLP-1RAs in the eye were searched until April 2025, including thorough cellular studies, animal studies, and clinical observation surrounding glaucoma, diabetic retinopathy, and other ocular diseases. This review delineates the multifaceted mechanisms by which GLP-1RAs exert ocular benefits, including retinal ganglion cell (RGC) rescue via calcium channel and GABAergic modulation, mitochondrial protection through PINK1/Parkin-mediated mitophagy and ERK1/2-HDAC6 signaling, as well as potent anti-inflammatory effects via cytokine reduction and immune cell recruitment inhibition. Moreover, GLP-1RAs contribute to retinal remodeling by stabilizing vascular integrity and extracellular matrix homeostasis, and they display anti-angiogenic properties critical for mitigating neovascular complications. Clinical evidence underscores a reduced incidence of glaucoma and potential benefits in diabetic retinopathy. However, conflicting evidence highlights context-dependent risks, such as early DR worsening in poorly controlled glycemic states. In conclusion, this review synthesizes molecular insights and clinical evidence to underscore GLP-1RAs’ potential as multi-targeted disease-modifying agents, urging further research to translate preclinical promise into safe, effective ocular therapies.

## 1 Introduction

Glucagon-like peptide-1 (GLP-1) is an incretin hormone released from gut enteroendocrine cells and regulates blood sugar by increasing insulin while reducing glucagon secretion and inhibiting stomach emptying and food intake (Drucker, 2022). However, its effects extend far beyond glycemic control, a diversity that arises from the widespread distribution of its receptor. Beyond its primary location in pancreatic β-cells, GLP-1 receptor (GLP-1R) has been identified in various other tissues, including the gastrointestinal tract, lung, kidney, heart, bone, central nervous system, liver, and adipose tissue (Bu et al., 2024). Recent studies have highlighted the presence of GLP-1R in mice retinal endothelial cells, with diabetic conditions leading to downregulation of these receptors (He et al., 2024). First characterized in the late 1990s for its role in enhancing β-cell insulin sensitivity (Edwards et al., 1999; Scrocchi et al., 1996), this mechanism propelled the development of GLP-1 receptor agonists (GLP-1RAs), initially approved for diabetes (2005) and later for obesity (2014) (Jastreboff and Kushner, 2023). Beyond metabolic control, emerging evidence reveals GLP-1RAs’ pleiotropic effects, including anti-inflammatory, antioxidant, and neuroprotective properties. These actions stem from their ability to suppress pro-inflammatory cytokines, mitigate oxidative stress by reducing reactive oxygen species (ROS), and inhibit neurotoxic astrocyte activation (Athauda and Foltynie, 2016; Ghosh et al., 2023). Such multifaceted benefits have expanded GLP-1RAs’ therapeutic scope to neurodegenerative, cardiovascular, and chronic kidney disease, etc. (Kristensen et al., 2019; Tang et al., 2024)

Ocular diseases, including glaucoma, diabetic retinopathy (DR), and age-related macular degeneration (AMD), impose a staggering global burden, with glaucoma alone affecting 95 million individuals and causing irreversible vision loss in over 10 million (Jayaram et al., 2023). Current therapies, such as intraocular pressure (IOP)-lowering agents for glaucoma or anti-VEGF drugs for DR, primarily target symptoms rather than underlying neurodegeneration or chronic inflammation (Stein et al., 2021). Furthermore, these interventions often rely on single-pathway mechanisms, which do not account for the multifactorial pathogenesis of ocular diseases. In DR, for example, hyperglycemia-induced vascular damage is accompanied by neuroinflammation and oxidative stress; however, no approved therapy simultaneously targets these interconnected mechanisms. These limitations are further compounded by the lack of therapies for early disease stages, where neuroprotection or vascular stabilization could halt progression.

**FIGURE 1:**
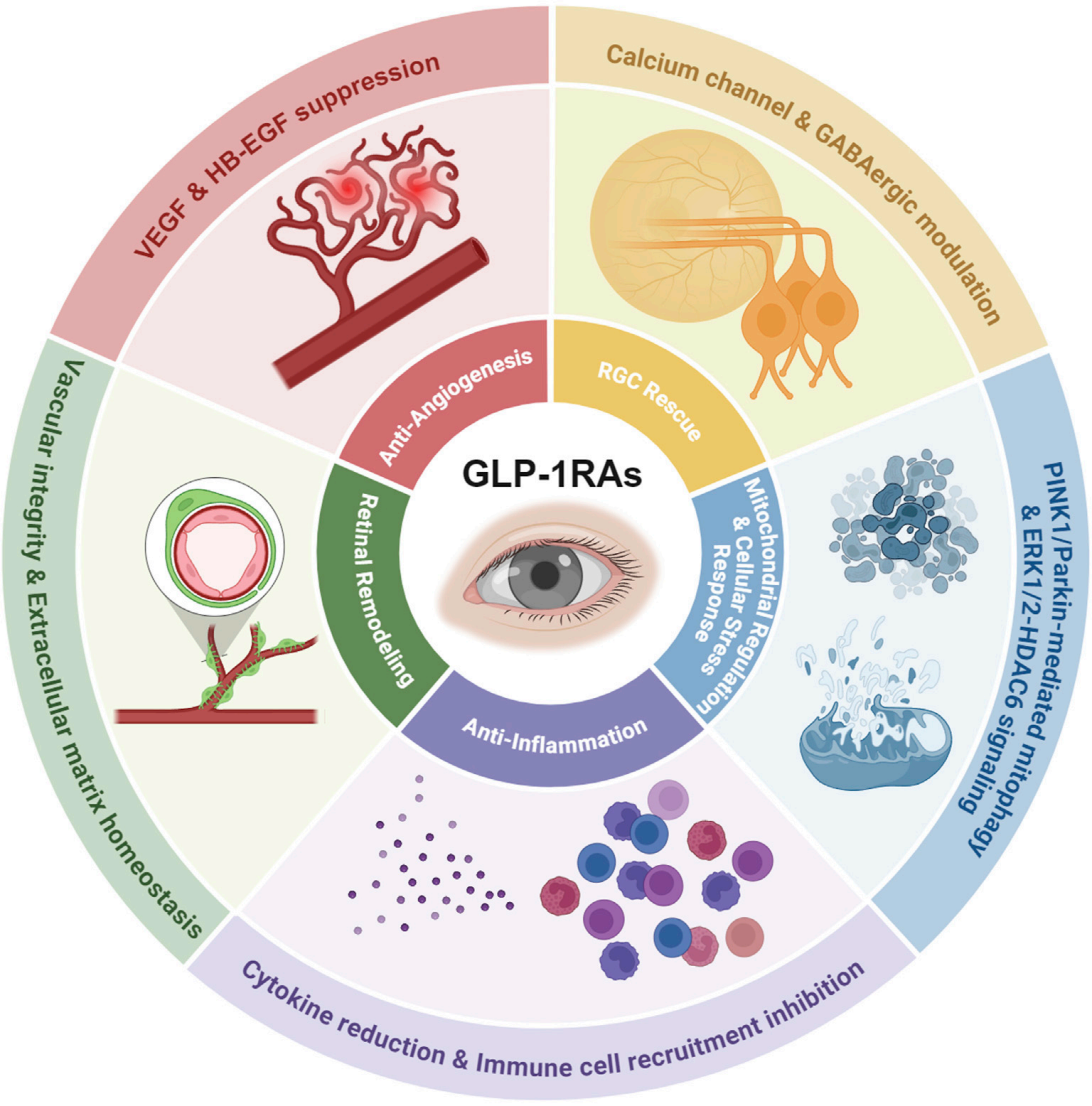
The mechanisms of GLP-1 RAs’ effects on the eye. As summarized and discussed in the following review, the pleiotropic effects of GLP-1RA on ocular therapy include RGC rescue via calcium channel and GABAergic modulation, mitochondrial protection through PINK1/Parkin-mediated mitophagy and ERK1/2-HDAC6 signaling, anti-inflammatory effects via cytokine reduction and immune cell recruitment inhibition, retinal remodeling ability by stabilizing vascular integrity and extracellular matrix homeostasis, and anti-angiogenic properties critical for mitigating neovascular complications.

Emerging evidence positions GLP-1RAs as a transformative candidate for ocular therapeutics. Unlike conventional approaches that target isolated symptoms or pathways, GLP-1RAs simultaneously tackle the multifactorial etiology of ocular diseases through their pleiotropic mechanisms. Preclinical studies demonstrate their capacity to reduce retinal ganglion cell apoptosis in diabetic models (Wang et al., 2023). Clinically, observational data suggest a 19% lower glaucoma incidence in GLP-1RA-treated diabetic patients than those on other antidiabetic regimens (Niazi et al., 2024), hinting at the disease-modifying potential. As illustrated in Figure 1, this review comprehensively analyzes the mechanistic basis of GLP-1RAs in ocular diseases, synthesizes emerging clinical and preclinical evidence, and underscores their potential to redefine therapeutic strategies for vision-threatening disorders through multi-targeted disease modification.

## 2 Molecular mechanism of GLP-1RA in the eye

Hernández et al. (2016) reported the simultaneous existence of GLP-1 and GLP-1R in the human retina for the first time. Predominantly located in the ganglion cell layer of the retina with additional sparse expression detected in the inner nuclear layer and outer nuclear layer, GLP-1R was found to be expressed at high levels in the retina, even surpassing in tissues like the liver and bowel. The study also found that GLP-1R expression was similar in the retinas of both diabetic and non-diabetic individuals, though GLP-1 levels, associated with neuroprotective ability, were significantly lower in diabetics. This established the presence of a potentially new target for therapeutic intervention in ocular diseases with a focus on neurodegeneration in the retina.

At the same time, as the pleiotropic effects of GLP-1RAs are gradually exhibited, their molecular mechanism, besides lowering blood glucose, is gaining increasing attention. In recent years, more research has examined the possible molecular mechanism of the drugs in the eye, hoping to facilitate understanding of their connection with ocular diseases and assist future therapeutic development.

### 2.1 RGCs’ rescue

**FIGURE 2:**
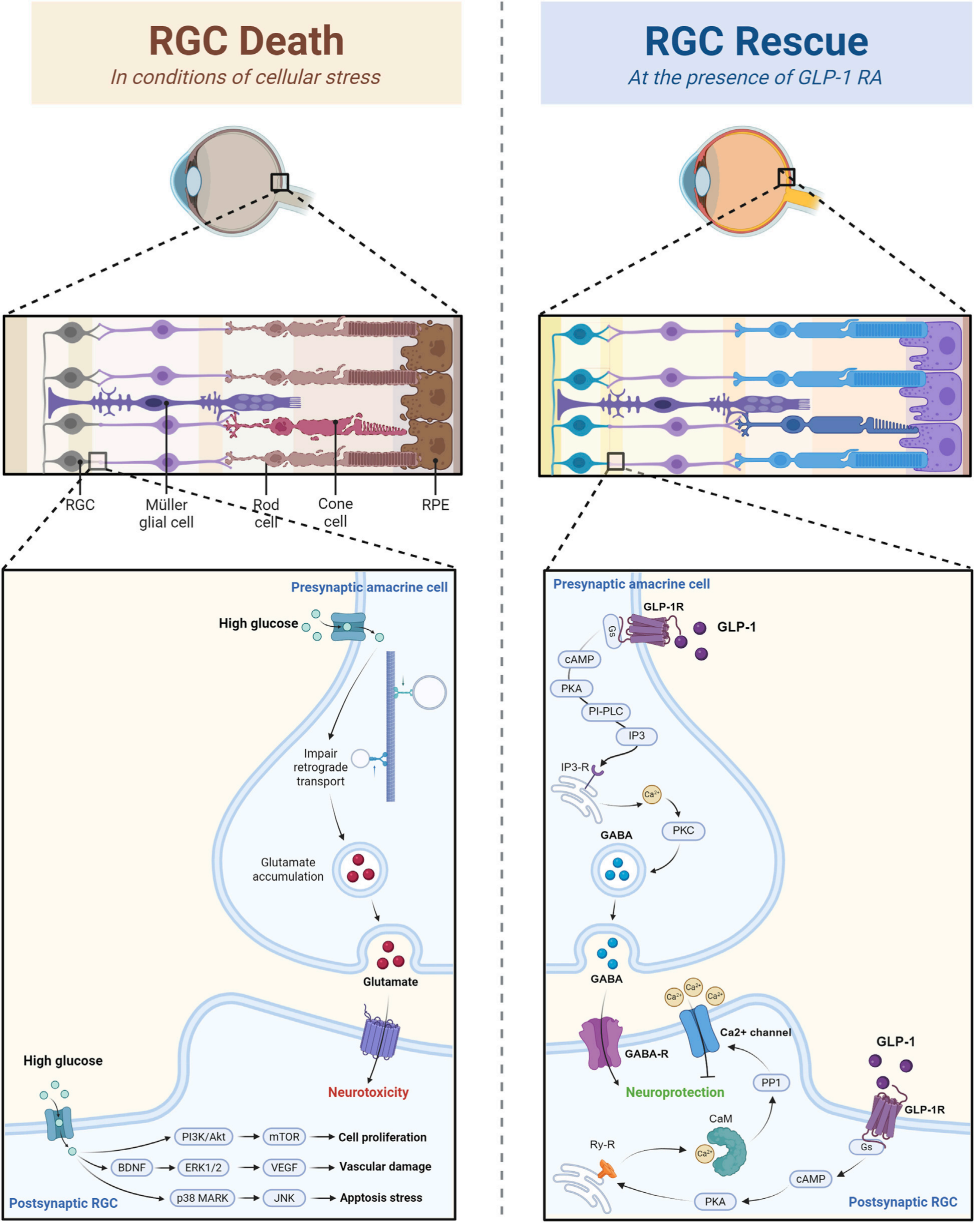
The rescuing effect of GLP-1 RAs on retinal ganglion cells through calcium channel and GABAergic modulation. The figure illustrated that in conditions of high glucose and cellular stress, RGC death is induced by the glutamate-mediated neurotoxicity and altered gene expression. However, GLP-1RA can inhibit calcium influx in postsynaptic RGC and enhance GABA release in presynaptic amacrine cell, and thus prevent calcium overload and excitation-induced RGC damage.

Notable RGC loss is reported under chronic diabetic situations. High glucose impairs retrograde axonal transport and leads to glutamate accumulation, which induces neurotoxicity. It also activates p38 MARK, ERK1/2 cascade, and JNK cascade, which promotes apoptosis and inflammation (Potilinski et al., 2020). One of GLP-1RA’s most dominant benefits on eye diseases is its direct effect on RGCs’ function, rescue, and survival. According to current studies, the molecular pathways involved in GLP-1RAs’ impact on RGCs’ rescue can be categorized into two groups (Figure 2).

#### 2.1.1 Calcium channel modulation

Recent research has shown that exendin-4, a GLP-1RA, suppresses L-type voltage-gated calcium channels (L-VGCC) activity through a cascade involving cAMP/PKA, ryanodine receptors, and calmodulin (CaM). By inhibiting calcium influx, exendin-4 reduces the calcium overload, which leads to apoptosis and excitation-induced neurotoxicity in RGCs, promoting RGC survival and function in DR (Wang et al., 2023).

The significant impact of GLP-1RAs on calcium homeostasis and cellular behavior is also confirmed in other cell types apart from RGCs. In pulmonary vein cardiomyocytes (Chan et al., 2023), GLP-1 amide modulates ionic currents by decreasing L-VGCC and Na^+^/Ca^2+^ exchanger current while increasing sarcoplasmic reticulum Ca^2+^ content. Mediated through PKA, CaMKII, and Na^+^/Ca^2+^ exchanger signaling pathways, GLP-1RAs reduce pulmonary vein automaticity and arrhythmogenesis, ultimately suppressing atrial fibrillation initiation. This finding shed light on the shared pathogenesis of ocular and cardiovascular diseases and provided hope for broader clinical application of GLP-1RAs besides its initial glucose-lowering ability.

However, GLP-1RAs’ effects on calcium channel modulation are multifunctional. For instance, liraglutide has been shown to enhance intracellular calcium levels in aortic endothelial cells, activating the CaMKKβ/AMPK axis to phosphorylate eNOS and CREB. This signaling cascade ultimately attenuates vascular inflammation by downregulating pro-inflammatory mediators and inhibiting monocyte adhesion (Krasner et al., 2014). This bidirectional calcium modulation underscores the intricate crosstalk between GLP-1RA-mediated pathways. Such multifaceted regulatory networks, compounded by tissue-specific variations in calcium handling, substantially complicate the therapeutic targeting of GLP-1RAs.

#### 2.1.2 GABAergic modulation

In diabetic rats, GABAergic transmission is disrupted, leading to excitation-induced RGC damage. Shao and colleagues discovered that GLP-1 administration restores the balance of inhibitory signaling by enhancing GABA release, thereby promoting RGC survival and improving visual function in early DR. More specifically speaking, GLP-1 receptor activation facilitates the presynaptic release of GABA into RGCs via the Gs/cAMP/PKA/Phospholipase C (PLC)/IP3/Ca2+/CaM signaling pathway, ultimately increasing the GABAergic miniature inhibitory postsynaptic currents (m-IPSCs) frequency and contrast sensitivity (Shao et al., 2024).

The GABAergic modulation ability of GLP-1RAs is more thoroughly studied in brain tissues and cells. In the striatal tissue of the 3-nitro propionic acid rat model, Sayed et al. (2020) observed that vildagliptin, a DPP-4 inhibitor, elevated GLP-1 levels and subsequently activated the PI3K/Akt signaling pathway. Specifically, GLP-1 activated the PI3K/Akt cascade through phosphorylation, enhancing CREB activation and promoting the expression of BDNF and other neurotrophic factors. The resulting increase in GABA release helps restore the balance of excitatory and inhibitory neurotransmission by decreasing glutamate activity, promoting neuronal survival, and enhancing mitochondrial function. Although no clinical trials have examined the impact of GLP-1RAs on Huntington’s disease (HD), more and more evidence in animal models is looking promising (Duarte et al., 2018).

The hippocampus is recently reported to be at the center of the GLP-1 pathway network. Cadmium-induced neurotoxicity, reflected by decreased Aβ42, p-tau, acetylcholine, and GABA in the hippocampus along with increased toxic glutamate, triggers cognitive impairment and Alzheimer-like changes. Recent studies have found that increased level of GLP-1 in the hippocampus restores GABA and reverses this neurotoxic effect by reducing oxidative stress through the SIRT1/Nrf2/HO-1 pathway, promoting autophagy through the AMPK/mTOR pathway, and alleviating hippocampal apoptosis by inactivating pro-apoptotic kinase GSK-3β (Arab et al., 2023). Clinical evidence and real-world data from three large cardiovascular trials (LEADER, SUSTAIN 6, and PIONEER 6) have also supported the beneficial effect of GLP-1RAs in dementia (Nørgaard et al., 2022). Ongoing clinical trials evoke and assess oral semaglutide’s clinical effectiveness and disease-modifying impacts in early-stage Alzheimer’s disease (AD) (Cummings et al., 2025).

The anorectic effects of GLP-1 can also be explained by its GABAergic modulation in the nucleus tractus solitarius (NTS) cells. When liraglutide binds to GLP-1R on GABAergic neurons in the NTS, it directly potentiates GABA release and suppresses food intake (Fortin et al., 2020). The neuroprotective effect of GLP-1RAs through GABAergic modulation not only supports its clinical potential in neurodegenerative diseases such as AD, HD, and Parkinson’s diseases (PD), but also adds to our fundamental understanding of GLP-1’s pleiotropic molecular network with a special focus on its ocular therapeutic potential.

### 2.2 Mitochondrial regulation and cellular stress response

As a premise, we know that the pathology of many neurodegenerative diseases and eye diseases are interconnected. For example, the elevated IOP in glaucoma eyes exerts mechanical stress at the optic nerve head, a key site for RGC damage, and leads to neurodegeneration and visual impairment. Metabolic stress, calcium dysregulation and neuroinflammation mediated by glial cells like astrocytes and microglia further exacerbates RGC loss (Bou Ghanem et al., 2024). Animal studies also discovered the neuroprotective effects of neurosteroids, allopregnanolone and its enantiomer, in treating glaucoma by activating GABA receptors and thus preventing apoptotic cell death of RGC (Ishikawa et al., 2022; Ishikawa et al., 2021). Although not yet directly examined in the eyes of human patients, these shared pathology encourage researchers to draw lessons from GLP-1RAs’ success in neuroprotection and transfer it into treating eye diseases.

**FIGURE 3:**
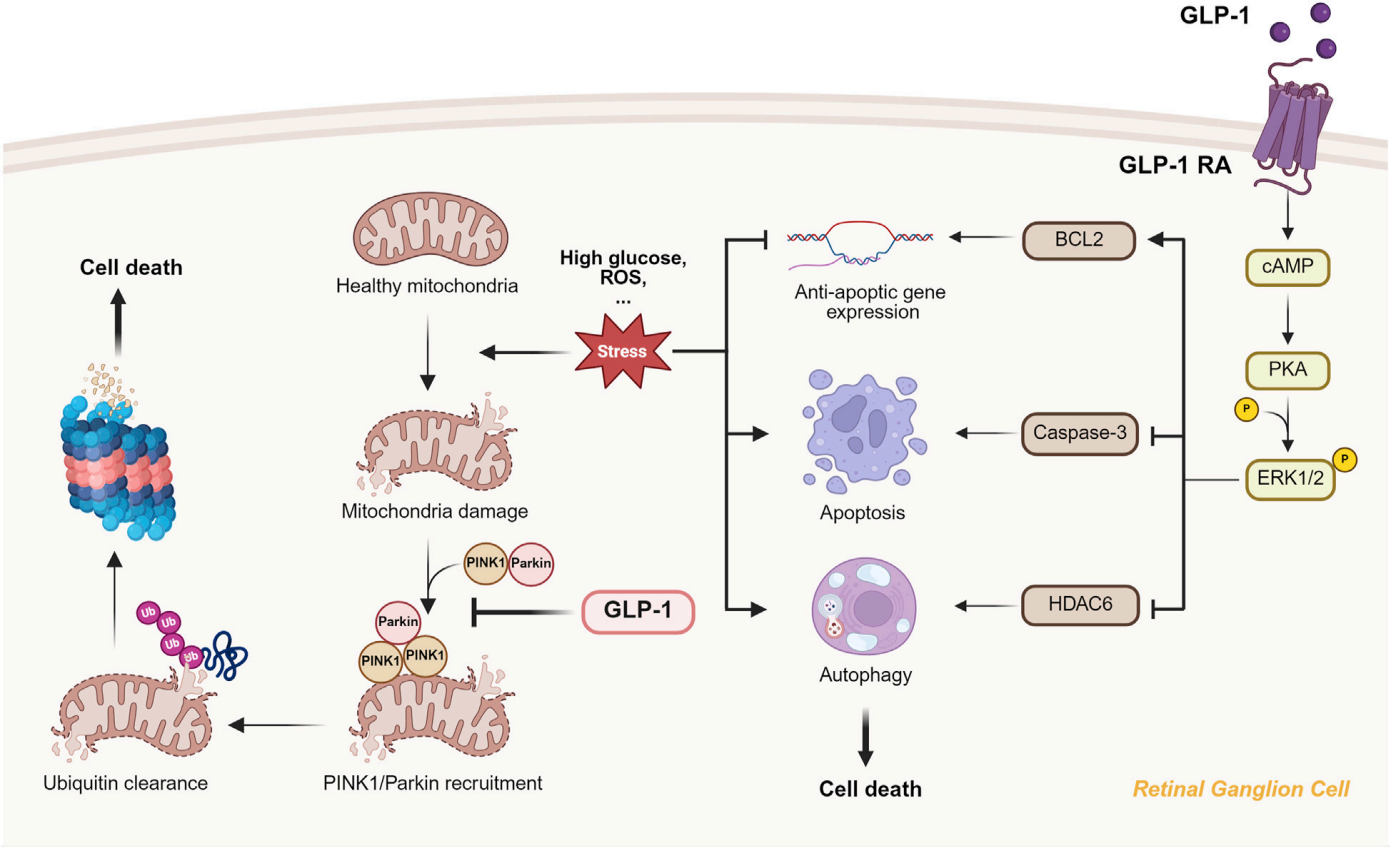
The mitochondrial protection and oxidative stress suppression provided by GLP-1 RAs through PINK1/Parkin-mediated mitophagy and ERK1/2-HDAC6 signaling. The figure showed that GLP-1RA can inhibit PIN1/Parkin accumulation on the damaged mitochondria and prevent subsequent ubiquitin clearance and RGC death. What’s more, binding of GLP-1RA to RGC activates cAMP/PKA/ERK1/2 pathway, which further inhibit anti-apoptic gene expression, apoptosis and autophagy through different molecular pathway.

In many eye diseases, oxidative stress and mitochondrial dysfunction are key contributors to RGC degeneration. According to our current understanding of GLP-1RAs’ mitochondrial regulatory effect and oxidative stress suppression ability on eye diseases, the molecular pathways mainly involve the PINK1/Parkin-mediated mitophagy pathway and the GLP-1R-ERK1/2-HDAC6 signaling pathway (see Figure 3). These two pathways are connected by focusing on mitochondrial homeostasis and the cellular response to stress, specifically oxidative stress and mitochondrial damage. Both pathways protect retinal cells (including RGCs) from damage induced by high glucose or ischemic conditions.

#### 2.2.1 PINK1/Parkin-mediated mitophagy

Under normal conditions, PINK1 (PTEN-induced kinase 1) is continuously degraded in healthy mitochondria. However, when mitochondria become damaged or depolarized due to oxidative stress or high glucose levels in DR, PINK1 accumulates on the outer mitochondrial membrane. It recruits Parkin for downstream ubiquitin clearance of the damaged mitochondria, also known as mitophagy. Although mitophagy under normal physiological conditions helps regulate mitochondrial health, excessive mitophagy often induces cell death and neurodegenerative disorders. GLP-1RAs, such as liraglutide, inhibit the PINK1/Parkin pathway and restrain excessive mitophagy, protecting RGCs from a high glucose environment. This leads to neuroprotection of RGCs and helps preserve retinal function (Zhou H-R. et al., 2021).

#### 2.2.2 ERK1/2-HDAC6 signaling pathway

The GLP-1R-ERK1/2-HDAC6 signaling pathway complements the mitophagy pathway by modulating oxidative stress and autophagy. Upon activation by GLP-1RA, the receptor stimulates cAMP production, which activates the PKA pathway and modulates the ERK1/2 signaling cascade. In the context of DR, this leads to the phosphorylation of ERK1/2, increasing downstream expression of anti-apoptotic genes like BCL2 and reducing the activity of pro-apoptotic markers such as Caspase-3. The ERK1/2 pathway also regulates HDAC6, pivotal in microtubule dynamics control and autophagy. Through HDAC6 inhibition, the pathway promotes the removal of damaged cellular components, reducing oxidative stress and enhancing cellular repair mechanisms in retinal cells (Cai et al., 2017).

#### 2.2.3 Beyond the eye

In renal tubular cells (Wang et al., 2021) and brain tissue in animal stroke model (He et al., 2020), exenatide and liraglutide activate NAD^+^-dependent deacetylase SIRT1, reducing mitochondrial dysfunction and oxidative stress by decreasing ROS production, thereby preventing apoptosis induced by lipotoxicity. Novel studies found that GLP-1RAs such as liraglutide, semaglutide and exendin-4 reduces α-synuclein aggregation respectively in MPTP (a mitochondrial complex I inhibitor) and 6-OHDA induced PD mice models as well as rat model of α-synucleinopathy. It protects dopaminergic neurons from oxidative stressed DNA damageand alleviate key pathological features of PD including oxidative stress, mitochondrial dysfunction and neurodegeneration, backing up its therapeutic potential for neurodegenerative diseases (Lin et al., 2021; Bu et al., 2021; Zhang et al., 2019; Zhang et al., 2022). In astrocytes, GLP-1R signaling promotes fatty acid oxidation, maintains mitochondrial integrity, and supports glucose homeostasis. However, a lack of GLP-1R is also observed to trigger a stress response that boosts fibroblast growth factor 21 and elicits beneficial systemic effects such as memory improvement and glucose homeostasis (Timper et al., 2020). This apparent contradiction highlights the complex and adaptive nature of cellular signaling and compensatory mechanisms under oxidative stress and urges researchers to pay attention to the downstream regulation of GLP-1 (fibroblast growth factor 21 in this context) when developing GLP-1RAs medication.

In aging heart tissue, liraglutide is reported to activate IRS1/eNOS/PKG pathways, restoring mitochondrial function by improving mitochondrial membrane potential and reducing oxidative stress (Durak and Turan, 2023). Similarly, semaglutide ameliorates doxorubicin-induced cardiotoxicity by reducing BNIP3-mediated mitochondrial dysfunction through the PI3K/AKT pathway, enhancing mitochondrial function and reducing cardiac injury (Li et al., 2024), potentiating its clinical application in cardiovascular diseases.

As we can see, the role of mitochondria regulation and cellular stress response stands at the center of many diseases. By understanding how GLP-1RAs influence mitochondrial function and stress responses in the eye, researchers can identify pathways and biomarkers that are also relevant in the heart, brain or other organs, and *vice versa*, promoting broader therapeutic applications and potentially reducing the time and cost of developing treatments for complex, multi-organ diseases.

### 2.3 Anti-inflammation effect

**FIGURE 4:**
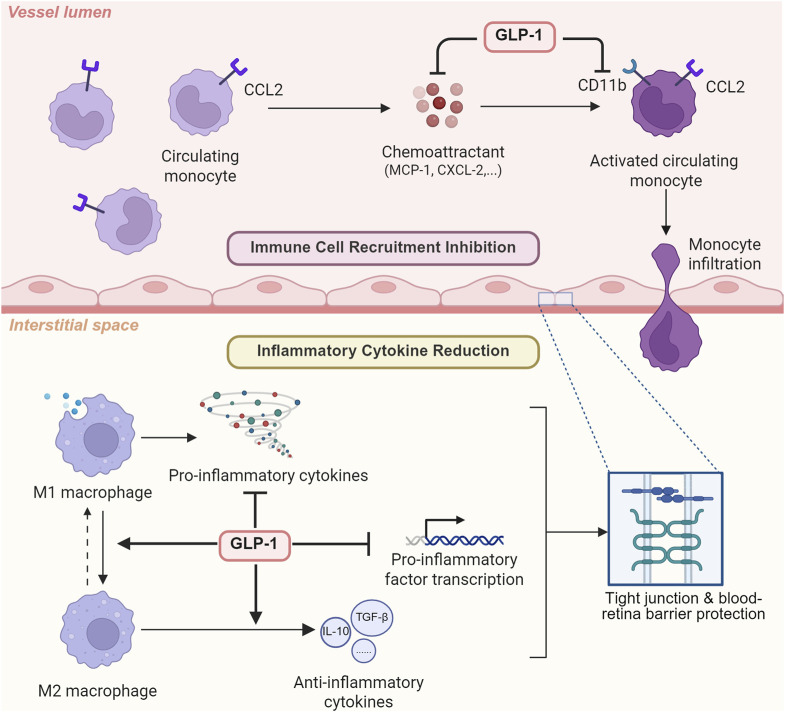
The anti-inflammatory effect of GLP-1 RAs via cytokine reduction and immune cell recruitment inhibition. To exert its anti-inflammatory effects, GLP-1RA can not only inhibit immune cell recruitment by suppressing chemoattractant and downregulating macrophage CD11b, but also directly reduce inflammatory cytokine expression.

The role of inflammation lies at the center of various eye diseases’ pathology. Microglia/macrophages (CD11b+ cells) respond to elevated IOP, the clinical feature of glaucoma, and produce three main pro-inflammatory cytokines: IL-1α, TNF-α, and C1q. These cytokines are sufficient and necessary for inducing both astrocyte transformation into neurotoxic A1 and blood-retina barrier destruction, resulting in the subsequent retinal ganglia cell death and various ophthalmopathy (Guttenplan et al., 2020; Liddelow et al., 2017). Apart from microglia/macrophages, Müller cell activation and gliosis are observed in animal models of glaucoma and diabetic retinopathy. As a subtype of glial cells vital for retina structural support, cellular homeostasis maintenance, and blood-retina barrier preservation, its reactivation would decrease l‐glutamic acid uptake, which results in excitotoxicity, as well as sabotage the integrity of the blood-retina barrier, leading to ultimate retinal ganglia cell death and vision damage (Ren et al., 2020). The anti-inflammation effect of GLP-1RAs is shown in Figure 4.

#### 2.3.1 Cytokine reduction

Besides the glucose homeostasis controlling function of GLP-1RAs, its cytokine-reducing ability is gradually recognized, and thus, its potential therapeutic effect on eye diseases is starting to gain attention. In muscle tissue of aged mice, lowered level of pro-inflammatory markers IL-1β, TNF-α, and IL-6 was observed after GLP-1RA dulaglutide treatment (Khin et al., 2021; Nguyen et al., 2020). Although the beneficial role of liraglutide in osteoarthritis has long been acknowledged, it was not until recently that a study hypothesized this GLP-1RA mediated macrophage transition to an M2 anti-inflammatory phenotype, inhibiting pro-inflammatory cytokine release in a dose-dependent manner (Meurot et al., 2022). Other animal studies examined the specific molecular pathway involved in exendin-4’s immune-suppressive effect. Yusta et al. (2015) discovered that exendin-4 treatment in Glp1r+/+ mice elevated cAMP levels in isolated intraepithelial lymphocytes while reducing the transcription of multiple inflammatory mediators, including IL-1β, IL-6, IL-22, IL-12β, TNF-α, CCL2, CXCL1, and CXCL2. These lowered cytokines level is proved in multiple animal glaucoma models to be capable of protecting tight junctions, preventing blood-retina barrier breakdown and ultimately rescuing retinal ganglia cells (Fan et al., 2014; Guttenplan et al., 2020), which strongly suggest the anti-inflammatory effect of GLP-1RAs on eye diseases.

#### 2.3.2 Immune cell recruitment inhibition

Apart from inflammatory cytokines secretion reduction, inhibited macrophage infiltration and immune cell recruitment also played a vital role in GLP-1RAs’ anti-inflammatory ability. Detailed mechanisms and molecular pathways were studied in cell and animal models. Through downregulating monocyte chemoattractant protein-1 expression, semaglutide treatment, an FDA-approved GLP-1 RA medicine targeting Type II diabetes mellitus (T2DM) (Christou et al., 2019), inhibited monocyte migration and infiltration from the circulatory system to inflammatory regions (Pontes-da-Silva et al., 2022). Atherosclerosis-model mice treated with exendin-4, another GLP-1RA, also demonstrated reduced circulating levels of monocyte chemoattractant protein-1, C-X-C motif chemokine ligand-1 (CXCL-1), and ultimately neutrophil recruitment (Wang et al., 2014). More specifically speaking, the mechanism potentially involved downregulation of macrophage CD11b, which played a vital role in monocyte adhesion, as well as decreased expression of macrophage modulators including CXCL-10, CXCL-1, C-C motif chemokine ligand-1 (CCL-2) and tissue inhibitor of metal protease-1 (TIMP-1) (Robinson et al., 2015). Given that immune cell accumulation plays a vital role in eye diseases’ pathogenesis, previous researchers have also studied the effect of GLP-1RAs on macrophages and discovered that it could reduce macrophage infiltration in addition to altering macrophage phenotypes in multiple sclerosis and nephropathy (Chiou et al., 2019; Lee and Jun 2016).

### 2.4 Retinal remodeling

**FIGURE 5:**
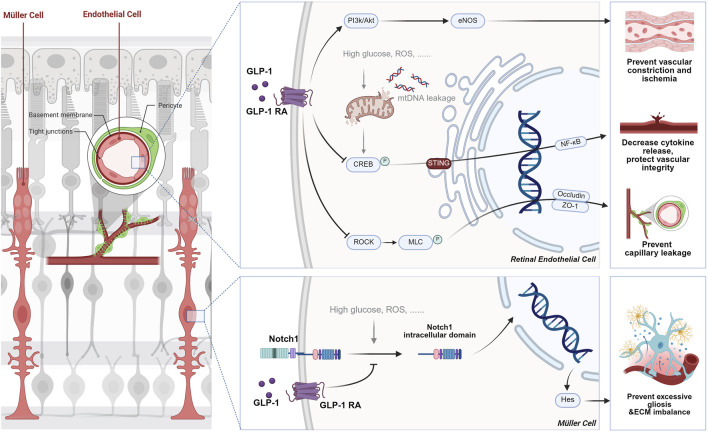
The role of GLP-1 RAs in retinal remodeling by stabilizing vascular integrity and extracellular matrix homeostasis. In vascular remodeling, GLP-1RAs enhance endothelial function by inhibiting the RhoA/ROCK signaling pathway, which reduces endothelial cell cytoskeletal breakdown and albumin leakage in DR. Additionally, they activate the PI3K-Akt-eNOS pathway, promoting nitric oxide production that helps regulate capillary tone and restores microvascular perfusion. In terms of ECM remodeling, GLP-1RAs prevent the activation of the Notch1-Hes1 pathway, reducing gliosis and inflammation in Müller cells, thus promoting ECM homeostasis and preventing fibrosis in retinal diseases like DR.

Recent discoveries have revealed the remodeling capabilities of GLP-1RAs in the retina. The following four molecular pathways collectively describe how GLP-1RAs facilitate retinal remodeling, primarily by enhancing vascular function and restoring ECM homeostasis. Each pathway targets specific aspects of vascular and cellular integrity, contributing to the overall protection and remodeling of the retinal vasculature in conditions like DR and ischemic retinal diseases (see Figure 5).

#### 2.4.1 Vascular remodeling

In DR, the RhoA/ROCK signaling pathway is activated by high glucose, which leads to endothelial dysfunction, breakdown of the blood-retinal barrier (BRB), and capillary leakage. This occurs through the phosphorylation of myosin light chain (MLC) by ROCK, resulting in cytoskeletal reorganization and changes in endothelial cell adhesion. Wei and colleagues (Wei et al., 2022) recently discovered that GLP-1 receptor activation inhibits this signaling pathway by modulating ROCK activity, leading to a reduction in the phosphorylation of MLC, which stabilizes the endothelial cell cytoskeleton and tight junctions by upregulating the expression of tight junction proteins such as Occludin and ZO-1. This ultimately decreases albumin leakage into the retina, thus improving the vascular permeability and preventing the capillary degeneration and pericyte loss typically seen in DR.

Besides the GLP-1R-ROCK-p-MLC signaling pathway, the PI3K-Akt-eNOS-NO-cGMP pathway has also been vital in retinal capillary tone and microvascular patency. The PI3K-Akt pathway is a well-established signal transduction pathway that regulates various cellular processes, including metabolism, survival, and vascular function. In retinal endothelial cells, activation of the GLP-1 receptor increases PI3K/Akt signaling, which activates endothelial nitric oxide synthase (eNOS), leading to NO production. This potent vasodilator is critical in regulating capillary tone and maintaining vascular patency. Zhai’s team (Zhai et al., 2020) found that exendin-4, a GLP-1 receptor agonist, enhances eNOS activity and NO production, which helps relax retinal capillaries and restore microvascular perfusion in ischemia-reperfusion (I/R) injury models. By improving capillary blood flow and microvascular tone, this pathway aids in vascular remodeling by preventing vascular constriction and capillary collapse, which are common in retinal diseases like DR and ischemic retinopathy.

Last but not least, inflammation modulation also contributes to vascular protection. Stimulator of interferon genes (STING) is a key player in innate immune responses, typically activated by cytosolic DNA. In DR, mitochondrial DNA leakage due to cellular stress or damage activates STING, which promotes inflammation and vascular dysfunction in retinal endothelial cells. Activation of STING triggers a signaling cascade involving TBK1, IRF3, and NF-κB, leading to the production of pro-inflammatory cytokines and further endothelial damage. GLP-1RAs suppress the activation of the STING pathway by reducing the levels and nuclear translocation of CREB. This enhances mitochondrial function and reduces mitochondrial DNA leakage, resulting in decreased inflammatory cytokine release and improved vascular integrity in the retina (He et al., 2024).

#### 2.4.2 ECM remodeling

Recent advances in research on eye disease pathogenesis have highlighted the significance of the ECM’s standard structure and function. The Notch1-Hes1 signaling pathway regulates cell differentiation, proliferation, and apoptosis. Translocation of Notch1’s inner cellular domain into the nucleus subsequently activates the transcription of Hes1. As a transcriptional suppressor, Hes1 in the retina inhibits the differentiation of specific cell types and leads to excessive gliosis, increased inflammatory cytokine release, and ECM imbalance. In eye diseases like DR, high blood glucose and oxidative stress activate the Notch1-Hes signaling pathway, leading to ECM imbalance and promoting vascular dysfunction, inflammation, and retinal damage (Fan et al., 2020). A significant discovery is that liraglutide, a GLP-1RA, inhibits the activation of Notch1. This action prevents gliosis in Müller cells and helps restore ECM homeostasis. Treatment with liraglutide decreases the levels of pro-inflammatory cytokines, such as TNF-α, and reduces gliosis markers, like GFAP, in Müller cells. This improvement promotes ECM remodeling and helps prevent the pathological fibrosis associated with various eye diseases (Shan et al., 2023).

### 2.5 Anti-angiogenic effect

Abnormal angiogenesis plays a significant role in the pathogenesis of several eye diseases, particularly those involving retinal ischemia and neovascularization. Improper blood vessels are prone to leak, bleed, and scar, leading to vision loss and other retinal complications. Several studies have investigated the anti-angiogenic effect of GLP-1RAs in ischemic retinopathies and choroidal neovascularization.

Zhou L. et al. (2021) examined the molecular pathways in GLP-1R modulation of microglia. Their work describes how microglial activation can directly promote angiogenesis by secreting VEGF and other proangiogenic cytokines in ischemic retinopathies. When GLP-1R is activated, microglial activation is suppressed, reducing the levels of VEGF and other cytokines involved in neovascularization. GLP-1R activation also inhibits NF-κB activation through the cAMP/PKA signaling pathway, suppressing inflammatory and angiogenic gene expression in retinal cells, including VEGF.

In the context of wet age-related macular degeneration (AMD), geniposide (GEN), a GLP-1RA, is found to downregulate HB-EGF (heparin-binding epidermal growth factor) secretion from retinal pigment epithelium (RPE) cells under hypoxic conditions through the miR-145-5p/NF-κB signaling axis. GEN enhances the expression of miR-145-5p, which inhibits the NF-κB pathway and thus suppresses inflammatory responses and angiogenesis (Gu et al., 2021).

## 3 Clinical impact of GLP-1RAs on the eye

The promising mechanism of GLP-1RAs in the eye further facilitates clinical evidence. Despite a few potential ocular risks caused by GLP-1RAs, more clinical studies continue to discover and confirm their beneficial role in the eye, providing hope for creating a new weapon to treat eye diseases.

### 3.1 Glaucoma

Studies examining the effect of GLP-1RAs on glaucoma risk in diabetes patients is a research hotspot that emerged in recent years. Separate retrospective cohort studies carried out by Sterling et al. (2023) and Chuang et al. (2024) in different populations both exhibited significantly lower incidences of open-angle glaucoma in diabetic patient groups treated with GLP-1RAs medication compared with the control group. More specifically speaking, Sterling’s group observed a decreased percentage of newly diagnosed glaucoma after GLP-1RAs exposure from 1.33% to 0.51%, and Chuang’s research discovered lowered glaucoma onset with a significant adjusted hazard ratio (aHR) (0.712, 95% CI: 0.533–0.936. P = 0.0025). More recent research has also looked into GLP-1RAs’ beneficial influence on glaucoma but focused on this effect at different time ranges. The nationwide, nested case-control study carried out by Niazi et al. (2024) observed a strong correlation between GLP-1RAs treatment with a 19% decreased likelihood of glaucoma onset, which was particularly notable in patients receiving the agonists for periods extending beyond 3 years. However, another study found a significantly lowered risk of developing glaucoma and ocular hypertension in all patient groups receiving the treatment at 1, 2, and 3 years (41%, 50%, and 41% risk reduction, respectively) (Muayad et al., 2024). The controversy surrounding whether a shorter treatment duration could lead to significant protection results may stem from the fact that the earlier study primarily enrolled patients in second-line diabetes treatments. This approach could reduce the dosage of individual drugs, such as GLP-1RAs, compared to the latter study. A recent retrospective cohort also observed that, even in overweight patients without diabetes, the risk of primary open-angle glaucoma and ocular hypertension were significantly lower in the GLP-1RA group at both 3 and 5 years of follow-up (Vasu et al., 2025), suggesting the benefit of GLP-1RAs in the eye as an independent factor apart from blood glucose control.

Apart from this, previous studies have also examined the specific influence of GLP-1RAs on IOP changes, RGCs’ survival, etc. Hallaj and colleagues discovered that exposure to GLP-1RAs was strongly associated with reduced IOP in diabetic patients after glaucoma surgery (Hallaj et al., 2024). Another study by Lawrence’s team confirmed the effect of GLP-1RA drugs on RGCs’ rescue in hypertensive mouse models of glaucoma (Lawrence et al., 2023). The findings strongly added to the potential benefit of GLP-1RAs in reducing glaucoma risks. They urged researchers to dive deeper into the mechanism behind this correlation to better understand glaucoma pathology, hoping to develop novel management approaches toward the disease.

### 3.2 Diabetic retinopathy

The number of trials and research examining the relationship between GLP-1RAs and DR is growing rapidly in recent years. A meta-analysis in 2023 examined 93 clinical trials from ClinicalTrials.gov that compared GLP-1RAs’ direct or indirect impact on DR with placebo, insulin, or oral antidiabetic medications (Kapoor et al., 2023). The results suggested that the use of GLP-1 RAs was notably linked to a higher risk of early-stage DR and early retinal adverse events when compared to placebo. However, when compared to insulin, GLP-1 RAs provided protection against advanced-stage DR. What’s more, its demographic analysis showed significant differences between the GLP-1 RA and comparator groups in factors such as age, HbA1c, body weight, BMI, duration of diabetes, sex, race, and so on, suggesting that the effect of GLP-1RAs on DR is yet controversial and that it may vary based on the specific GLP-1RA used, as well as the patient’s demographic and clinical characteristics.

A Swedish cohort study examined the impact of multiple GLP-1RAs on the incidence of DR in 2,390 diabetic patients. It matched patients treated with GLP-1RAs with those who did not use the medication and found that GLP-1RAs were associated with a significantly lower risk of DR, with a hazard ratio (HR) of 0.42. Mendelian randomization analysis also showed an inverse relationship between GLP-1 receptor expression and severe DR, indicating the potential of GLP-1RAs in reducing the risk of DR in type 2 diabetes patients (Zheng et al., 2023). However, the SUSTAIN six trial evaluated semaglutide, a GLP-1 analog, in patients with type 2 diabetes and high cardiovascular risk and discovered that semaglutide was associated with a higher incidence of diabetic retinopathy complications compared to placebo, with a hazard ratio (HR) of 1.76, particularly in patients with pre-existing DR (Vilsbøll et al., 2018). This suggests that semaglutide may cause early worsening of DR, especially in patients with poor glycemic control, highlighting the importance of monitoring DR during GLP-1 RA therapy. Apart from trials supporting the two opinions mentioned above, other results denied the possible impact of GLP-1RAs on DR. The EXSCEL trial (Bethel et al., 2020) and LEADER trial (Marso et al., 2016) investigated the effect of exenatide and liraglutide, respectively, on microvascular and cardiovascular outcomes in patients with type 2 diabetes. Despite the drugs’ cardiovascular benefits, both trials found no significant difference in retinopathy outcomes between the GLP-1RAs and placebo groups.

This seemingly controversial impact of GLP-1RAs on DR may be because most trials focused on microvascular and cardiovascular outcomes rather than DR. Thus, the direct influence of GLP-1RAs on DR still calls for future investigation.

### 3.3 Other ocular events

Apart from glaucoma and DR, the association between GLP-1RAs and other ocular events has also been investigated in recent years. Hao et al. (2022) observed promising therapeutical effects of SM102, a novel GLP-1RA, on hyperglycemia, weight control, and diabetic dry eye syndrome via its antioxidant properties. A cohort study by Fan’s team (Fan et al., 2023) also confirmed a significantly reduced risk of dry eye syndrome, superficial keratitis, and infectious keratitis in diabetic patients treated by GLP-1 compared with placebo. A pathology research observed inflammation, fibrosis, reduced tear secretion and decreased level of GLP-1R in the lacrimal gland of diabetic dry eye. Liraglutide restored proper autophagic flux by alleviating the accumulation of P62 (an autophagy dysfunction marker) in the lacrimal gland, reduced oxidative damage by enhancing clearance of ROS and promoting cellular repair mechanisms, and potentially addressed dry eye syndrome (Sun et al., 2025). What’s more, a recent retrospective, multicenter cohort study denied higher occurrence of nonarteritic anterior ischemic optic neuropathy (NAION) in GLP-1RAs users and suggested that its potential benefits for blood glucose control and cardiovascular health likely outweigh its risks (Chou et al., 2025), despite that a few research have reported association between semaglutide prescription and a higher risk of NAION(61). Regarding age-related macular degeneration (AMD), a recently published retrospective cohort study reported that GLP-1RAs were associated with reduced hazard of non-exudative and exudative AMD compared to metformin, insulin and statins (Allan et al., 2025). Although no mechanism hypothesis has yet been posed upon GLP-1RAs’ impact on most adverse ocular events, these primary findings still require that clinical workers monitor GLP-1RA users’ eye condition. The information of current clinical studies and trials studying GLP-1RAs’ effect on various eye diseases is summarized in Table 1.

**TABLE 1:** Current clinical studies and trials studying GLP-1RAs’ effect on various eye diseases.

Author	Year	Disease	Drug	Main research outcome	ClinicalTrials.gov identifier/PMID
Sterling	2023	POAG, glaucoma suspect, low-tension glaucoma	NLY01	Reduced incidence of open-angle glaucoma in diabetic patients	34413054
Chuang	2024	OAG	Exenatide, liraglutide	Reduced risk of glaucoma in diabetic patients	38250602
Niazi	2024	POAG	Exenatide, liraglutide, lixisenatide	Reduced glaucoma onset with long-term GLP-1RA treatment (over 3 years)	38490274
Muayad	2024	POAG	Exenatide	Reduced risk of glaucoma and ocular hypertension after 1, 2, and 3 years of GLP-1RA treatment	39182626
Hallaj	2024	glaucoma	Exenatide, albiglutide, dulaglutide, lixisenatide, semaglutide, liraglutide	Reduced IOP in diabetic patients’ post-glaucoma surgery	39237049
Zheng	2023	Severe NPDR	Liraglutide, semaglutide, dulaglutide	Lowered severe NPDR risk	36737746
Vilsbøll	2018	NPDR	Semaglutide	Increased incidence of DR complications in patients with pre-existing NPDR	NCT01720446 (Phase III, completed)
Bethel	2020	DR	Exenatide	No significant difference between exenatide and placebo	NCT01144338 (Phase III, completed)
Marso	2016	DR	Liraglutide	No significant difference between liraglutide and placebo	NCT01179048 (Phase III, completed)
Hao	2022	dry eye	SM102	Improved hyperglycemia, weight control, and reduced diabetic dry eye symptoms	35184645
Fan	2023	dry eye and keratitis	Exenatide, liraglutide	Reduced risk of diabetic dry eye syndrome, superficial keratitis, and infectious keratitis	37893823
Chou	2025	NAION	Semaglutide	No significant difference of NAION occurrence between semaglutide and placebo	39491755
Hathaway	2024	NAION	Semaglutide	Increased risk of NAION associated with semaglutide prescription	38958939
Allan	2025	Non-exudative and exudative AMD	Dulaglutide, lixisenatide, albiglutide, exenatide, semaglutide, liraglutide	Reduced risk of both non-exudative and exudative AMD compared to metformin, insulin and statins	39863057

Abbreviations: AMD, age-related macular degeneration; DR, diabetic retinopathy; GLP-1RA, glucagon-like peptide-1 receptor agonist; IOP, intraocular pressure; NAION, nonarteritic anterior ischemic optic neuropathy; NPDR, nonproliferative diabetic retinopathy; POAG, primary open-angle glaucoma; RGC, retinal ganglion cell.

## 4 Future challenges

### 4.1 Mechanistic validation across ocular pathologies

Despite progress in understanding molecular pathways such as calcium channel modulation and mitochondrial mitophagy, the relative contributions of these mechanisms to specific diseases remain unclear. GLP-1RAs inhibit RGC apoptosis in glaucoma through GABAergic signaling, but their role in AMD, characterized by oxidative stress and choroidal neovascularization, needs further investigation (Shao et al., 2024). Additionally, most mechanistic studies rely on animal models, raising concerns about translatability to humans due to interspecies differences in GLP-1R distribution and signaling (Drucker, 2022). Human retinal organoids and patient-derived cell lines could bridge this gap by providing disease-relevant platforms for mechanistic interrogation.

The latest diabetic retinopathy preferred practice guidelines from the American Academy of Ophthalmology indicate that the rising use of GLP-1RAs may lead to quick glycemic control and severe hypoglycemia and has been reported to cause higher rates of retinopathy complications, especially in patients with early-stage DR (Lim et al., 2025). Thus, the clinical application of GLP-1RAs on ocular diseases still requires caution and further research.

### 4.2 Delivery optimization

Systemic administration of GLP-1RAs reduces their availability in the retina and can lead to off-target effects, particularly gastrointestinal issues. Therefore, localized delivery methods, such as intravitreal implants, nanoparticle carriers, or gene therapy aimed at retinal GLP-1R expression, are essential for maximizing therapeutic effectiveness while minimizing systemic exposure. Recent advancements in sustained-release formulations, like PLGA nanoparticles for delivering exendin-4, show promise in preclinical models but still require validation in human studies (Wang et al., 2023).

### 4.3 Synergy with existing therapies

Given the promising potential of GLP-1RA in ocular therapeutics, combing it with other current medicine could shed light on novel therapeutic approaches. Sampedro’s team (Sampedro et al., 2019) discovered in mice that treatment with GLP-1RAs resulted in a significant decrease in VEGF levels in the retina, kidneys, and systemic circulation. This finding correlates with reduced tissue damage and improved vascular integrity, and also suggests a synergistic effect with current ocular medications, such as anti-VEGF drugs. A retrospective study explored the impact of prior GLP-1RA treatment on the response of DR patients to subsequent intravitreal anti-VEGF injections and other conventional ophthalmic therapy (Michaeli et al., 2024). The results demonstrated that patients who had previously received GLP-1RA treatment had a more favorable response to intravitreal injections, with improved visual acuity outcomes and reduced central retinal thickness. Additionally, the study highlighted that the need for repeated anti-VEGF injections was modestly lower in the GLP-1RA group, indicating a potential priming effect that enhances the efficacy of conventional ocular treatments. However, since the clinical efficacy of GLP-1RA drugs in ocular diseases is a newly emerging area of research, direct clinical evidence and higher-level trials are still lacking. Combining GLP-1RAs with anti-VEGF agents or IOP-lowering drugs may enhance efficacy but pose unresolved challenges. In DR, GLP-1RAs’ vascular stabilization effects (via RhoA/ROCK inhibition) (Wei et al., 2022) could complement anti-VEGF therapy, yet their interaction in modulating angiogenesis remains unexplored. Administering GLP-1RAs with neuroprotective agents like brimonidine in glaucoma also requires careful dose optimization to prevent additive side effects.

### 4.4 Long-term safety and off-target effects

Emerging reports link GLP-1RAs to rare ocular adverse events, including NAION (Hathaway et al., 2024). It is still uncertain whether these risks are caused by direct mechanisms such as changes in blood flow or are influenced by confounding factors like preexisting vascular conditions. Longitudinal registries and post-marketing surveillance studies are essential for assessing long-term eye safety, particularly in older populations with multiple health issues.

## 5 Conclusion

In conclusion, the expanding body of evidence supports that GLP-1RAs offer a multifaceted approach to ocular therapeutics by targeting key molecular pathways involved in retinal neuroprotection, mitochondrial regulation, inflammation, vascular remodeling, and angiogenesis. These agents target the symptoms of ocular disorders like glaucoma and DR while addressing the underlying pathogenic mechanisms, paving the way for potential disease modification. Despite promising findings from preclinical and observational clinical studies, challenges persist. Key issues include the need for mechanistic validation in human models, optimizing localized delivery to enhance retinal bioavailability, and ensuring long-term safety. Future research should focus on integrating advanced retinal models, synergistic treatment regimens, and rigorous clinical trials to harness the therapeutic potential of GLP-1RAs fully.

## Abbreviations

AD, Alzheimer’s disease; DR, diabetic retinopathy; GLP-1, glucagon-like peptide-1; GLP-1R, glucagon-like peptide-1 receptor; GLP-1RAs, glucagon-like peptide-1 receptor agonists; HD, Huntington’s disease; NAION, nonarteritic anterior ischemic optic neuropathy; NTS, nucleus tractus solitarius; PD, Parkinson’s disease; RGCs, retinal ganglion cells; ROS, reactive oxygen species; T2DM, type II diabetes mellitus.
